# Supplementary material for: TaKLU Plays as a Time Regulator of Leaf Growth via Auxin Signaling
Source: Int J Mol Sci. 2022 Apr 11;23(8):4219. doi: 10.3390/ijms23084219 (PMC9033062; doi:10.3390/ijms23084219)
Supplement: Supplementary file 1 [file ijms-23-04219-s001.zip › ijms-1654635-supplementary.pdf]

***TaKLU* Plays as a Time Regulator of Leaf Growth via Auxin Signaling**

Supplementary Material

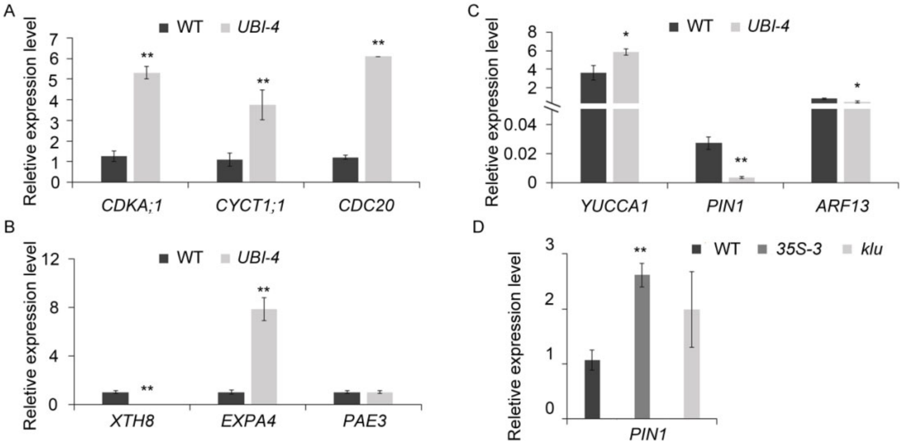

Supplementary Figure S1 Quantitative Real-time PCR analysis of the expression level of related genes in wheat and *Arabidopsis*. (A-C) Analysis of the expression levels of cell cycle (A), cell wall (B) and auxin (C) related genes in transgenic wheat (*UBI-4*) leaves. (D) Analysis of the expression levels of auxin (D) related gene in transgenic *Arabidopsis* (*35S-3*) leaves. All samples were taken from seedling leaves of different genotypes plants. Bars indicate SE (n > 3). Asterisks (*) and (**) indicate significant differences from their WT at P<0.05 and P<0.01 (t-test), respectively.

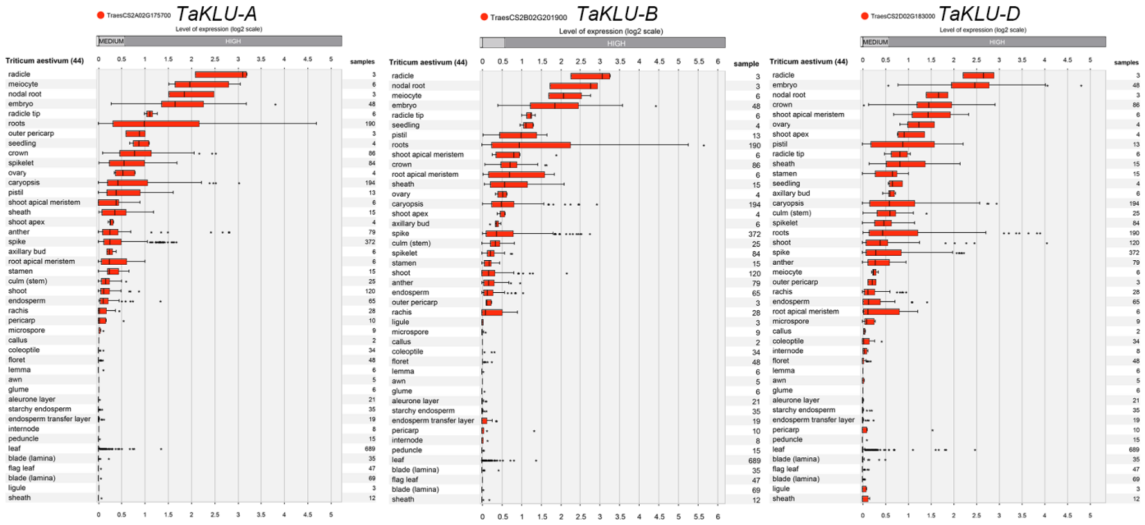

Supplementary Figure S2 *TaKLU* organization expression pattern analysis using the public network databases. *TaKLU* organization expression pattern analysis using the Genevestigator software (https://www.bits.vib.be/software-overview/genevestigator).

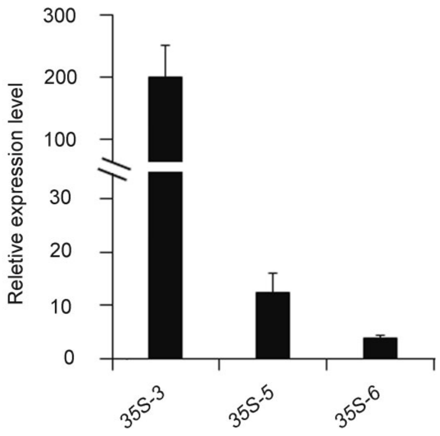

Supplementary Figure S3 The expression level of *TaKLU* in *Arabidopsis* was analyzed by Quantitative Real-time PCR. All samples about

Arabidopsis were taken from lateral inflorescences of different genotypes plants.

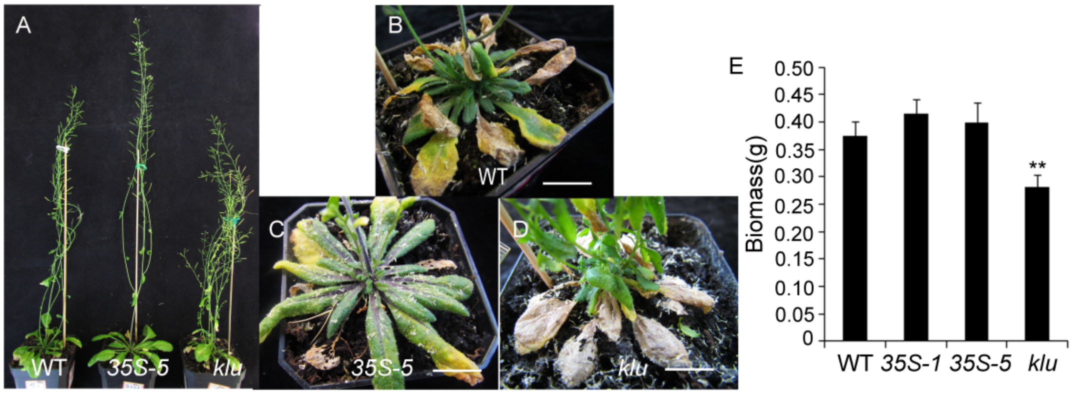

Supplementary Figure S4 *KLU* activity affects the apical dominance and longevity in *Arabidopsis*. (A) WT, *35S::TaKLU* (*35S-5*) and *klu* mutant plants at 70 days after germination. (B-D) Comparison of the leaf senescence of different genotypes at 70 days after germination. (E) Statistical analysis of biomass between different genotypes *Arabidopsis* (n > 7). Plants grown in the same environment were harvested and used for biomass determination after them completely dry. Bars indicate SE. Size bars represent 2 cm (B-D). Asterisks (*) and (**) indicate significant differences from their WT at P<0.05 and P<0.01 (t-test), respectively.

Supplementary Table S1 Primers used for Vector Construction

Primers' name	Sequence 5' to 3'
TaKLU-cDNA-F (*Xba* I)	TGCACGCGTCGATCTTTCCATGGTTACCGG
TaKLU-cDNA-R (*Bgl* II)	AGAAGATCTCGAGCCACGGCCCTGCAGA
pAtKLU-F (*Eco*R I)	ACGGAATTCTGGTCTGGCTTTAGGACGATC
pAtKLU-F (*Xba* I)	TGCACGCGTCTCAGGCTGAGTTATGGCAG

Supplementary Table S2 Primers used for *TaKLU* analyses

Primers' name	Sequence 5' to 3'
TaKLU-RT-F	GACATGATCGCCGTCCTTTG
TaKLU-RT-R	ACGATGCACTGGATGTAGGG
UBC-F	CTGCGACTCAGGGAATCTTCTAA
UBC-R	TTGTGCCATTGAATTGAACCC
ACTIN-F	AAATCTGGCATCACACTTTCTAC
ACTIN-R	GTCTCAAACATAATCTGGGTCATC

TaYUCCA1-RT-F	TGGATCGATTCATCCTGGCG
TaYUCCA1-RT-R	GTCACCTCCTTCACTGCTCC
AtPIN1-RT-F	GGCATGGCTATGTTCAGTCTTGGG
AtPIN1-RT-R	ACGGCAGGTCCAACGACAAATC
TaPIN1-RT-F	GGACCCGAACAACAATGGGA
TaPIN1-RT-R	CAGACGAGCGACCAGATGAG
TaARF13-RT-F	AGATCTCCGAGACGACCCTT
TaARF13-RT-R	GAAGAGGGCAGCTCAGTGAG
AtCDKA;1-RT-F	ACTGGCCAGAGCATTCGGTATC
AtCDKA;1-RT-R	TCGGTACCAGAGAGTAACAACCTC
TaCDKA;1-RT-F	TGGCCAGGTGTTTCTTCGTT
TaCDKA;1-RT-R	GGCAGTTCGGGCATTGATTC
AtCYCT1;1-RT-F	AGGATGGTAGGCGGTTATGTCAGG
AtCYCT1;1-RT-R	AAGCAAAGCGACGTCCTCAGAC
TaCYCT1;1-RT-F	GCACCATCCTTACAAGCCCT

TaCYCT1;1-RT-R	CCAAGCCTTCTCACCGTCAT
AtCDC20-RT-F	GGATTCTGCATCCAACCGTCAAC
AtCDC20-RT-R	TCCATGCCAGTGATCCTACTCTTG
TaCDC20-RT-F	ATGGCCATACTTCCCGTGTC
TaCDC20-RT-R	TCGGAGCATCAGGAAACACA
AtXTH8-RT-F	TCTATCGCAGCAACACCGACAC
AtXTH8-RT-R	TGCTTTGTCTGAAATCCACATCCG
TaXTH8-RT-F	GTTCAACACTGACGGCACC
TaXTH8-RT-R	CCTGTCCAGAAGCGTCGTAT
AtEXPA4-RT-F	GCAGGGTGCCATGTAGAAAGAGAG
AtEXPA4-RT-R	TCATCCAACCAGTCCGTGATCC
TaEXPA4-RT-F	TTCCTCCACATCGCGCAGTA
TaEXPA4-RT-R	AGGTTGAAGTAGGAGTGGCC
AtPAE3-RT-F	TATGACGCTTGGCAGGTACAAG
AtPAE3-RT-R	TCCAAGAGCCGCTTAGGTCAAC

TaPAE3-RT-F	CAATCGTCTAGCCTGCAACG
TaPAE3-RT-R	CATTCCAAGTAGCCGGGACC
